# Trace Elements and Ferritin in Pig Saliva: Variations during Fattening, Time of Sampling, Effect of Dirtiness and Stability under Different Storage Conditions

**DOI:** 10.3390/antiox12030649

**Published:** 2023-03-05

**Authors:** Alba Ortín-Bustillo, Damián Escribano, Silvia Martínez-Subiela, Asta Tvarijonaviciute, Alberto Muñoz-Prieto, Marina López-Arjona, José J. Cerón, Fernando Tecles

**Affiliations:** 1 Interdisciplinary Laboratory of Clinical Analysis (Interlab-UMU), Regional Campus of International Excellence ‘Campus Mare Nostrum’, University of Murcia, Campus de Espinardo s/n, 30100 Murcia, Spain; 2 Department of Animal Production, Regional Campus of International Excellence ‘Campus Mare Nostrum’, University of Murcia, Campus de Espinardo s/n, 30100 Murcia, Spain; 3 Department of Animal and Food Science, Universitat Autònoma de Barcelona, 08193 Bellaterra, Spain

**Keywords:** saliva, pig, fattening cycle, diurnal, variations, dirtiness, stability, biomarkers, sampling

## Abstract

The objective of this study was to evaluate the possible changes of zinc (Zn), copper (Cu), iron (Fe) and ferritin during the entire productive cycle in fattening pigs and at different diurnal sampling times. Moreover, the possible effects of the presence of pen contaminants and storage stability at different temperature conditions were assessed. The analytes changed along the different phases of the fattening productive cycle, showing, in general, higher values at the initial phases. In addition, statistically significant variations were found in Zn and Cu measurements at different sampling times of the day. In the spectrophotometric assays, the values of all analytes significantly increased after adding high concentrations of feces or feed. However, when low concentrations of feces or feed were added, only Cu showed a significant increase. Overall, the salivary levels of Zn, Cu, Fe and ferritin in pigs can change during different fattening phases and the different hours of the day. These analytes were more stable at −80 °C and, if saliva is contaminated with feces or feed, it can lead to an increase in these analytes.

## 1. Introduction

The application of the saliva as a non-invasive sample for analysis has grown more popular in recent years [1,2]. Saliva has a wide spectrum of components [3], with potential as biomarkers to assess health status [4]. Collecting saliva samples is a non-invasive and easy way to perform sampling without the need to acquire specialized skills [5,6]. In veterinary science, the use of saliva implies an easier collection in less time and, therefore, more efficient sampling of a higher number of animals [7]. The use of saliva is particularly convenient in porcine species, where blood sampling is stressful for the animal and technically complicated due to its thick layer of fat body, lack of easily accessible superficial vessels and susceptibility to develop a state of stress [8].

Sialochemistry is a term that defines a series of laboratory tests performed on saliva and includes biomarkers that provide diverse information on the health status of the animals [2]. Trace elements such as iron (Fe), zinc (Zn) and copper (Cu) are related to iron deficiency [9,10,11], inflammation and activation of the immune response among other physiological pathways [12,13,14,15]; they can be measured in porcine saliva by automated commercial assays [16]. Ferritin is a biomarker related to Fe that participates in several cellular functions [17] and it has a role in inflammation [18].

In a previous report, an analytical validation was performed for Fe, Zn, Cu and ferritin measurement in porcine saliva. These analytes were measured in saliva of Fe supplemented and non-supplemented piglets, with the supplemented group having higher values of ferritin and Zn in saliva [16]. Although the measurement of these analytes in the saliva of pigs is still not widely used, it could be of interest for the evaluation of different physiological processes in which these analytes are involved, for example, to evaluate the addition of different Cu levels to diet [19], the effect of Zn in the stress and immune response [20] or Fe in intestinal inflammation [21].

The aim of this study was to gain knowledge about the measurements of Zn, Cu, Fe and ferritin in the saliva of pigs. For this purpose, we evaluated whether these analytes could change under different physiological conditions, such as the entire productive cycle in fattening pigs and different sample times during the day. In addition, the effect of pen feces and feed contamination, and also the stability of the storage of these salivary analytes, were assessed in order to evaluate possible variations in the measurements due to these factors.

## 2. Materials and Methods

### 2.1. Animals and Biomedical Ethics

Saliva samples from pigs (*Sus Scrofa domesticus*, Large-White) located at the Veterinary Teaching Farm of the University of Murcia (Guadalupe, Murcia, Spain), which is free of the porcine respiratory virus and reproductive syndrome, were used. Animals were accommodated under general commercial housing and conditions conforming to European Union guidelines (Directive 2010/63/EU1).

### 2.2. Sampling

Saliva collection tubes (Salivette, Sarstedt, Aktiengesellschaft and Co., Nümbrecht, Germany) and fragments (5 × 2 × 2 cm) of polypropylene sponges (Esponja Marina, La Griega E. Koronis, Madrid, Spain) were used for sampling as previously reported [22]. Animals were allowed to chew a fragment of sponge attached to a thin, flexible metal rod for 1–2 min or until thoroughly moistened. Then, the sponge was introduced in a Salivette tube and transported refrigerated to the laboratory (which took around 2 h) where they were centrifuged (3000× *g*, 10 min, 4 °C). The obtained volume of saliva was transferred to 1.5 mL tubes (Eppendorf Ibérica, Spain) to be stored at −80 °C until analysis (less than a month in all cases). During sampling, animals that were drinking or eating were avoided. Dirty sponges or oral fluid with color were discarded.

### 2.3. Analysis of Zn, Cu, Fe and Ferritin

Fe, Zn and Cu were analyzed using colorimetric methods from BioSystems (Iron-Ferrozine, 11,509, BioSystems S.A., Barcelona, Spain) and Randox (Zinc, ZN2341, and Copper, CU2340, Randox Laboratories Ltd., Crumlin, UK). Ferritin was measured using an automated immunoturbidimetric assay (Ferritin latex, 31,935, Biosystems S.A., Barcelona, Spain). Chemistry analyzers (Olympus AU 400 for Zn, Cu and ferritin and Olympus AU 600 for Fe, Olympus Diagnostica GmbH1, Hamburg, Germany) were used for the analysis. The four methods were previously analytically validated for pig saliva samples, showing adequate precision and accuracy in a simple and fast manner, without using deproteinization pre-treatments in case of trace elements [16]. In that report, the intra- and inter-assay imprecision was <12% in average for all the assays, and the variation percentage compared with the values expected in the recovery tests was <9% [16].

### 2.4. Evaluation of Changes of Zn, Cu, Fe and Ferritin in Fattening Pigs during a Complete Productive Cycle

This was made in surplus samples from Large White pigs (24 males and 21 females) from 5 litters (average litter size of 10.0 ± 0.7) from a previous experiment [23]. Sampling was performed in the sucking phase at 24 days of life (T1); in the nursery phase one week after weaning (T2); at the end of nursery (T3); at the beginning of the growing-finishing phase after one week for acclimatization (T4); and at the end of the phase (T5).

### 2.5. Evaluation of Changes of Zn, Cu, Fe and Ferritin According to the Time of Sampling (Diurnal Variations Study)

This was made in surplus samples from a previous experiment [24]. A total of 40 Large White pigs (20 males and 20 females) were randomly chosen for the experimental study with an age around 5 months approximately, and they had ad libitum access to water and a commercial suitable diet. Saliva was sampled from the pigs at 0800 h, 1200 h, 1600 h and 2000 h within a same day. Pen temperature oscillated from 19 °C in the morning to 24 °C at midday.

### 2.6. Influence of Fecal and Feed Contamination of Saliva Samples in the Measurements of Zn, Cu and Fe

The experimental assay was performed based on a previous study made to evaluate the effect of pen feces and feed in salivary biomarker assays [25]. Clean saliva samples collected from apparently healthy finishing pigs (around 110 kg body weight and 5 months of age) were mixed to obtain pools (n = 16) of 12 mL. Pen feces and animals feed were also collected in falcon tubes (Eurotubo^®^, sterilized conic tubes 15 mL and 50 mL, Deltalab S.L, Barcelona, Spain). Feed was a commercial finisher diet (Cefusa, Murcia, Spain) integrated by corn, wheat, sunflower, barley, rapeseed and soybeans, nutritionally balanced (15.5% of crude protein -CP-; 0.79% standardized ileal digestible lysine, %SID Lys; and 13.5 MJ of metabolizable energy per kg -ME/kg-). It was given ad libitum in pellets with 400 μm particle size.

After that, five aliquots of each saliva pools were prepared and treated as follows: (1) control group (C group, n = 16) with 3.5 mL of saliva without any treatment; (2) effect of high pen feces contamination (HD group, n = 8) by adding 875 mg of pen feces, previously collected in a Falcon tube from the farm of the study, to 3.5 mL of clean pig saliva; (3) effect of low pen feces (LD group, n = 8), by adding 87.5 mg of pen feces to 3.5 mL of clean saliva; (4) effect of high feed contamination (HF group, n = 8) by adding 875 mg of commercial porcine feed, used at the farm of this study, to a 3.5 mL of clean saliva; (5) effect of low feed contamination (LF group, n = 8) by adding 87.5 mg of feed to 3.5 mL of clean pig saliva. For preparing those mixes, the pen feces or feed inside falcon tubes were crushed and mixed with the pools of saliva during 1 min and incubated at 38 °C for 5 min. Then, sponges were introduced in each falcon to absorb the mixture, placed in saliva collection tubes and centrifuged to obtain the pools.

The trace elements concentration of those mixtures was analyzed by the colorimetric methods, and also with ICP-MS (Agilent 7900 Inductively Coupled Plasma-Mass Spectrometry), to assess the possible effect of the method of measurement in the interferences. ICP-MS is a highly sensitive and accurate technique [26], previously validated for trace elements measurement in saliva [27], which requires a previous treatment of the samples with HNO_3_ 65% (500 µL of acid added to 500 µL of sample). As additional controls, the trace elements concentration of 87.5 mg of feces and of 87.5 mg of feed diluted in 3.5 mL of distilled water was previously measured by ICP. Concentrations were 245.55 ppb of Fe, 304.83 ppb of Cu and 723.82 ppb of Zn for the feces dilution; and 175.17 ppb of Fe, 731.67 ppb of Cu and 743.79 ppb of Zn for feed dilution. Therefore, the concentrations of each element were 10.07 µg of Fe, 15.24 µg of Cu and 29.68 µg of Zn per mg of feces; and 8.76 µg of Fe, 29.99 µg of Cu and 30.50 µg of Zn per mg of feed.

### 2.7. Storage Stability

The effect of different storage temperature conditions through time was evaluated for Zn, Cu, Fe and ferritin measurement in saliva of pigs. Ten saliva samples were collected from the Teaching Farm of the University of Murcia and were measured after collection (T0) and then, each sample was aliquoted in three subsamples: one was refrigerated at 4 °C, one frozen at −20 °C and one frozen at −80 °C. The samples were measured before storage (T0) and after 1 week (TW) and 6 months (T6). Percentages of loss and recovery of markers after each point of measurements were calculated as percentages from initial analysis (T0 as 100% of each analyte concentration of saliva sample). Measurements were calculated according to the following formula: (T − T0) × 100/T0 [28].

### 2.8. Statistical Analysis

All data were assessed for normality by the Shapiro–Wilk method. Since most of the data showed non-normal distribution, continuous variables were naturally log transformed prior to analysis by applying the formula ln (x + 1) for further analysis [29].

For the longitudinal and diurnal variation studies, a Mixed Linear Model of repeated measures was used in which time and sex were fixed factors and the individual was a random factor. Univariate analysis and Bonferroni post hoc test were further performed for any of those fixed factors considered as significant.

For assessing the effect of the presence of dirt in the saliva samples, the results obtained after the addition of feces or feed to the sample were analyzed by a General Linear Model (GLM) or repeated measures, followed by a Bonferroni post hoc test for pairwise comparisons.

For stability storage evaluation, the imprecision of the assay, calculated as the intra-assay coefficient of variation (Intra-CV), was used to assess stability. Thus, the biomarkers of this study were considered stable when changes observed in the stored samples did not exceed the significant change limit (SCL) acceptable for the assay, which was defined as SCL = 100% ± 2 × Intra-CV [30]. A two-way ANOVA test of repeated measures, followed by Dunnett’s multiple comparison test, was used to assess whether the percentage of change observed in analytes’ levels over time was statistically significant at different temperatures. Changes out of SCL and with significant differences from T0 were considered to have unacceptable stability for that storage conditions.

The calculations were made using GraphPad Prism 8 (GraphPad Software, San Diego, CA, USA) and SPSS (IBM SPSS Statistics for Windows, Version 28.0.1. IBM Corp, NY, USA) statistical packages, and the significant level was set at *p* < 0.05.

## 3. Results

### 3.1. Longitudinal Study

The results of the salivary analytes obtained at the different phases are shown in Table 1. Time effect was significant for all analytes.

Zn concentrations reached the highest values at the beginning of nursery. Then a progressive decrease was observed until they reached the lower values at the end of the growing phase.

Cu concentrations in saliva were lower at lactation and then significantly increased, reaching its peak at the beginning of nursery. Then Cu decreased in the growing phase. A significant gender effect was observed, with Cu concentrations being significantly higher in females than in males at the end of the nursery and growing phases.

Fe salivary concentrations were higher at lactation and the beginning of nursery, then they decreased by the end of nursery and values remained stable since then. Significantly higher Fe concentrations were detected in females than in males at the end of the nursery and growing periods.

Ferritin concentrations in saliva were higher at lactation, showing a strong decrease at the beginning of nursery. By the end of nursery, the ferritin concentrations achieved stable values until the end of the growing phase. Sex effect was significant since females showed higher values than males at nursery and by the end of growing phase.

In spite of the gender differences observed, the evolution of salivary biomarker concentrations was similar in both sexes since time–gender interaction was not significant in any case.

### 3.2. Diurnal Variations Study

**Figure 1:**
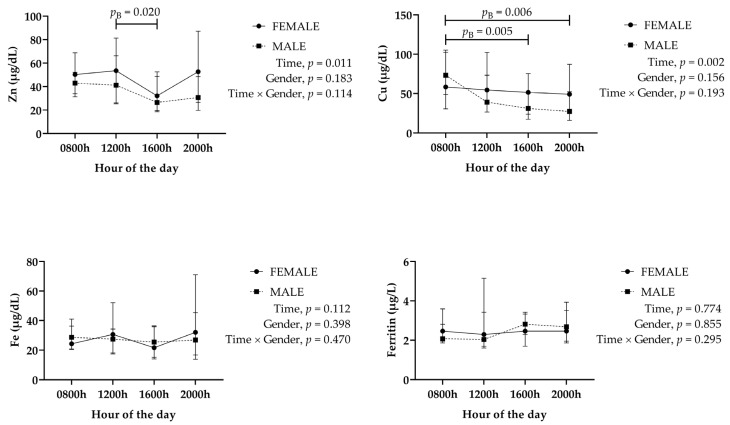
Results for the zinc (Zn), copper (Cu), iron (Fe) and ferritin concentrations obtained in saliva from 40 healthy fattening pigs (20 females, 20 males) at different hours of the day. *p*_B_: *p* value of the Bonferroni post hoc analysis.

The results of this section are shown in Figure 1. Time effect was significant for Zn and Cu salivary concentrations. Significantly lower Zn concentrations were observed at 1600 h compared with 1200 h. Cu concentrations were significantly lower at 1600 h and 2000 h compared with 0800 h. No gender effect was observed in Zn and Cu throughout the hours of the day. Fe and ferritin salivary concentrations remained stable along the day, with no significant changes due to time or gender.

### 3.3. Influence of Fecal and Feed Contamination

**Figure 2:**
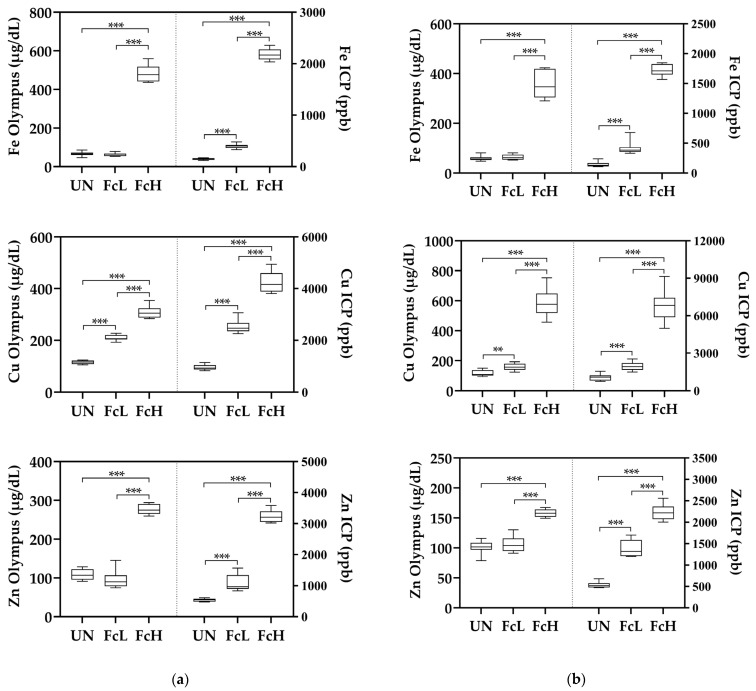
Results for the zinc (Zn), copper (Cu) and iron (Fe) after adding different quantities of pen feces (**a**) or feed (**b**) in pig saliva. Concentrations obtained in saliva pools without any treatment (UN—untreated)), after adding low quantity of pen feces or feed (FcL) and high quantity of pen feces or feed (FcH) by automated colorimetric assays and by ICP-MS method. *p*_B_: *p* value of the Bonferroni post hoc analysis. Asterisks indicate significant differences from the C group (***: *p* < 0.001).

The results of this section appear in Figure 2. The addition of high concentrations of feces or feed to the saliva samples significantly increased Zn, Cu and Fe values in both colorimetric and ICP methods (GLM *p* < 0.001 in all cases). No statistically significant changes were found in the colorimetric assays for Zn and Fe after the addition of low concentrations of feces or feed, whereas Cu was affected when the concentration of dirtiness was low. In the case of ICP assays, the presence of feces or feed at any concentration significantly increased the results in all analytes.

### 3.4. Storage Stability under Different Temperature Conditions

**Figure 3:**
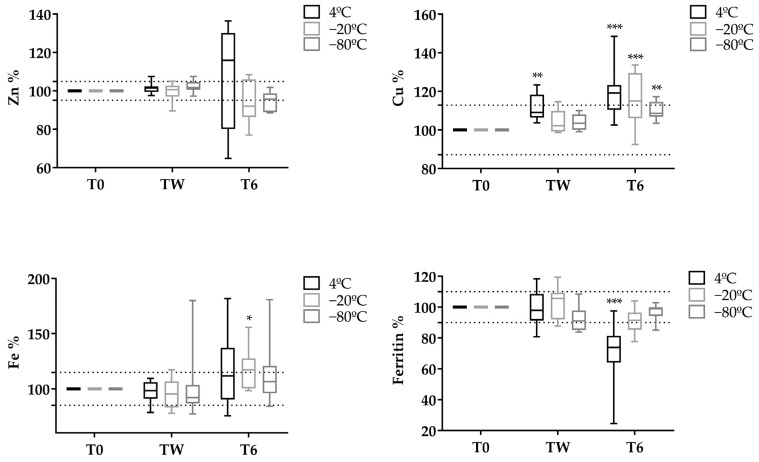
Stability plots of the zinc (Zn), copper (Cu), iron (Fe) and ferritin in ten porcine saliva samples kept at different temperatures. Results are expressed in percentages of the baseline concentration (fresh sample = 100% concentration). Each aliquot was stored at different temperatures and measured in fresh conditions (T0) and after 1 week (TW) and 6 months (T6). Dotted lines indicate the significant change limit (SCL) acceptable for the assay, which was defined as SCL = 100% ± 2 × Intra-CV. The plot shows median (line within box), 25th and 75th percentiles (box), 5th and 95th percentiles (whisker). Asterisks indicate significant differences from T0 (*: *p* < 0.05; **: *p* < 0.01; ***: *p* < 0.001).

Data obtained after evaluating stability of Zn, Cu, Fe and ferritin at different temperature storage conditions are shown in Figure 3. No statistically significant differences between the different storage conditions were observed in Zn after six months, but mean concentrations from samples stored at 4 °C were higher and showed higher variability than the frozen ones at this time, and saliva stored at −20 °C showed median levels out of SCL. In case of Cu, values were stable after one week at −20 °C and −80 °C. After six months, samples stored at −80 °C showed median values more similar to controls and within SCL. Fe was stable until six months of storage at −80 °C, with most of the values kept inside the SCL with no statistically significant changes at any time point. Ferritin showed a significant increase after six months of refrigeration with results out of SCL; whereas the values of frozen samples were maintained inside the SCL or nearby, with no statistically significant variation at any time point.

## 4. Discussion

In this report, the influence of different physiological conditions, such as the phases of fattening and other factors such as time of sample collection, sample contamination or type of storage, on the measurement of Zn, Cu, Fe and ferritin in pig saliva was studied. These data can contribute to the appropriate interpretation of these analytes and their better use in the evaluation of pig health and welfare.

In general, trace elements presented higher levels at the beginning of the fattening cycle, and their concentrations were lower in later phases. In the case of ferritin and Fe, the peak was presented in lactation. Values obtained at lactation in the analytes of this study were similar to those described in a previous paper [16]. The age could be the possible factor that causes these variations, in part, since it can influence the values of analytes in human saliva [31]. The decline of trace elements with age, which occurs in our report, has been described also in human serum [32,33]. In addition, different diet formulations in each production phase may play a role in the variations found in our experimental conditions.

In our study, the four different diurnal sampling times were selected to represent the times of sampling that could be taken in routine farm conditions [24,34]. We found significant changes depending on the time of sampling for Zn and Cu. Based on this, for these analytes, it could be recommended to sample the pig saliva in the morning or establish another reference range if sampling is performed in the afternoon, as has been suggested for other salivary analytes in pigs [24]. To our knowledge, there are no studies in which the analytes of our research have been measured at different times in other species; nevertheless, a similar decrease in Zn to that in our study has been described in human serum at 1500 h [35].

The evaluation of the effects of the presence of pen feces and feed, which can frequently contaminate saliva, indicated that the spectrophotometric assays used in the experimental conditions in our study are less affected by a low degree of contamination than the ICP-MS. The reason why ICP-MS was more affected by a low degree of dirtiness than the colorimetric assays should be further clarified. Among the possible reasons, it could be postulated that the perchloric acid digestion, or the form of complexes that could alter the dissociation constants of the trace elements, could have a possible influence on the results. On the other hand, at high contaminant concentrations, major changes and interferences were observed in all the analytes of our study, with both the spectrophotometric and ICP-MS giving falsely high values in a similar way as occurs with other salivary analytes in pigs [25]. 

In general, the levels of salivary biomarkers studied were more stable at frozen temperatures than at refrigeration. Moreover, samples stored at −80 °C were more stable during the experiment than at −20 °C. This lowest variability, found at −80 °C, has been reported for other salivary biomarkers in porcine samples [28]. Therefore, for measurements of salivary Zn, Cu, Fe and ferritin, it is highly recommended to store samples at −80 °C. More studies should be undertaken to delve into the reasons for the lower stability of the analytes in refrigeration or at −20 °C, testing if it could be related to the form of the complexes or due to action of the bacteria present in saliva that could be maintained in refrigeration.

As limitations of this report, it could be stated that the study was performed using samples from only one farm. Thus, it would be necessary to take precautions when extrapolating these results. Ideally, the possible retention of the sponge of the analytes in pig saliva should be evaluated. Since it was difficult to obtain saliva by flow in pigs, we tested the effect of the sponge in 10 samples of distilled water in which Fe, Cu and Zn, in the range of the concentrations found in porcine saliva samples, were added from the standards of the kits. The analytes were measured before and after mixing and spiked the with sponge and no significant differences were detected in the values of each analyte before and after the sponge was introduced (data not shown). So, it could be postulated that the sponge does not produce an evident interference in the analytes of our study. Moreover, the possible variations depending on other factors, such as the breed, age, season of the year or different productivity status, should be assessed in future studies, as well as changes due to diseases or stressful states. All these potential sources of variation should be considered in order to establish appropriate reference values for these analytes.

## 5. Conclusions

Concentrations of Zn, Cu, Fe and ferritin in pig saliva change with fattening, with most of them increasing at the suckling and beginning of nursery periods. In addition, Zn and Cu show variations throughout the hours of the day, having lower values at 1600 h. Moreover, falsely increased results can appear if samples are collected under high-dirt conditions or after being stored at inappropriate temperatures. Therefore, all these issues must be considered in the proper assessment of these measurements in porcine saliva.

## Figures and Tables

**Table 1:** Results obtained of salivary zinc, copper, iron and ferritin in 45 Large White pigs (21 females, 24 males) at suckling phase (T1); beginning (T2) and end (T3) of nursery phase; beginning (T4) and end (T5) of growing phase. Median (interquartile range) are expressed.

	Sampling Time						
Biomarkers	T1	T2	T3	T4	T5	Fixed Factors	*p* Value
Zn (µg/dL)	452.6 ^a^ (453.7)	2154.6 ^b^ (3104.3)	186.0 ^c^ (212.1)	170.3 ^c^ (258.1)	59.9 ^d^ (65.4)	Time	<0.001
Female	517.4 ^a^ (681.2)	2340.3 ^b^ (2040.7)	261.0 ^c^ (251.9)	172.5 ^c^ (208.2)	79.3 ^d^ (82.3)	Sex	0.102
Male	444.2 ^a^ (382.4)	1841.7 ^b^ (3328.4)	166.5 ^c^ (160.0)	170.0 ^c^ (288.4)	46.1 ^d^* (64.4)	Time * sex	0.189
Cu (µg/dL)	23.2 ^a^ (26.6)	194.8 ^b^ (185.8)	57.2 ^c^ (39.3)	73.6 ^c^ (94.5)	71.3 ^c^ (85.4)	Time	<0.001
Female	27.3 ^a^ (28.4)	182.2 ^b^ (248.5)	67.2 ^c^ (64.6)	81.9 ^c^ (95.6)	112.2 ^c^ (90.2)	Sex	0.038
Male	18.0 ^a^ (27.7)	195.7 ^b^ (151.9)	47.7 ^c^* (27.4)	64.1 ^c^ (99.6)	65.8 ^c^* (29.5)	Time * sex	0.203
Fe (µg/dL)	183.2 ^a^ (112.6)	133.2 ^ab^ (127.9)	46.7 ^d^ (30.7)	59.5 ^bc^ (128.0)	57.8 ^cd^ (50.5)	Time	<0.001
Female	160.8 ^a^ (130.2)	144.8 ^ab^ (139.8)	57.6 ^c^ (38.0)	58.5 ^bc^ (139.3)	74.2 ^bc^ (91.3)	Sex	0.046
Male	186.2 ^a^ (104.0)	98.2 ^ab^ (115.0)	46.4 ^c^* (31.6)	63.7 ^b^ (114.9)	49.6 ^c^* (38.9)	Time * sex	0.079
Ferritin (µg/L)	79.1 ^a^ (144.1)	8.3 ^b^ (15.6)	4.2 ^c^ (5.3)	3.0 ^c^ (5.0)	3.4 ^c^ (4.0)	Time	<0.001
Female	97.8 ^a^ (219.1)	16.1 ^b^ (25.7)	5.6 ^c^ (4.7)	2.9 ^c^ (5.4)	4.2 ^c^ (5.6)	Sex	<0.001
Male	60.0 ^a^ (101.4)	6.5 ^b^*** (8.8)	2.9 ^c^** (2.8)	3.1 ^bc^ (5.1)	1.6 ^c^* (3.1)	Time * sex	0.200

Zn: zinc; Cu: copper; Fe: iron; ferritin; BW: birth weight. Statistical analysis: a different letter indicates significant differences between sampling times; asterisks indicate significant differences between sexes (*: *p* < 0.05; **: *p* < 0.01; ***: *p* < 0.001).

## Data Availability

The data presented in this study are available on request from the corresponding author.
